# Whole-genome characterization and global phylogenetic comparison of cefotaxime-resistant *Escherichia coli* isolated from broiler chickens

**DOI:** 10.71150/jm.2412009

**Published:** 2025-04-29

**Authors:** Shahana Ahmed, Tridip Das, Chandan Nath, Tahia Ahmed, Keya Ghosh, Pangkaj Kumar Dhar, Ana Herrero-Fresno, Himel Barua, Paritosh Kumar Biswas, Md Zohorul Islam, John Elmerdahl Olsen

**Affiliations:** 1 Department of Veterinary and Animal Sciences, Faculty of Health and Medical Sciences, University of Copenhagen, Frederiksberg, Denmark; 2 School of Agricultural, Environmental and Veterinary Sciences, Faculty of Science and Health, Charles Sturt University, Wagga Wagga, New South Wales, Australia; 3 Department of Microbiology and Veterinary Public Health, Chattogram Veterinary and Animal Sciences University, Bangladesh; 4 Department of Analytical Chemistry, Nutrition and Bromatology, University of Santiago de Compostela, Spain; 5 CSIRO Health & Biosecurity, Australian Centre for Disease Preparedness, Geelong, Victoria, Australia

**Keywords:** antimicrobial resistance, *E. coli*, cefotaxime, one health

## Abstract

Antimicrobial resistance (AMR) poses a serious threat to public health, with the emergence of extended-spectrum beta-lactamases (ESBLs) in *Enterobacteriaceae*, particularly *Escherichia coli*, raising significant concerns. This study aims to elucidate the drivers of antimicrobial resistance, and the global spread of cefotaxime-resistant *E. coli* (CREC) strains. Whole-genome sequencing (WGS) was performed to explore genome-level characteristics, and phylogenetic analysis was conducted to compare twenty CREC strains from this study, which were isolated from broiler chicken farms in Bangladesh, with a global collection (n = 456) of CREC strains from multiple countries and hosts. The MIC analysis showed over 70% of strains isolated from broiler chickens exhibiting MIC values ≥ 256 mg/L for cefotaxime. Notably, 85% of the studied farms (17/20) tested positive for CREC by the end of the production cycle, with CREC counts increasing from 0.83 ± 1.75 log10 CFU/g feces on day 1 to 5.24 ± 0.72 log10 CFU/g feces by day 28. WGS revealed the presence of multiple resistance genes, including *bla*_CTX-M_, which was found in 30% of the strains. Phylogenetic comparison showed that the Bangladeshi strains were closely related to strains from diverse geographical regions and host species. This study provides a comprehensive understanding of the molecular epidemiology of CREC. The close phylogenetic relationships between Bangladeshi and global strains demonstrate the widespread presence of cefotaxime-resistant bacteria and emphasize the importance of monitoring AMR in food-producing animals to mitigate the spread of resistant strains.

## Introduction

Antimicrobial resistance (AMR) is a pressing global concern that threatens the efficacy of antibiotics and compromises our ability to combat infectious diseases. The imprudent use of antimicrobials in both human and veterinary medicine is a primary driver of this escalating crisis (Laxminarayan et al., 2013; Van Boeckel et al., 2017). Within the spectrum of AMR, extended-spectrum beta-lactamases (ESBLs) have emerged as a difficult challenge, particularly in the *Enterobacteriaceae* family. ESBLs, enzymes capable of hydrolyzing oxyimino-cephalosporins, confer resistance to critical antibiotics such as cefotaxime, ceftazidime, ceftriaxone, cefuroxime, and cefepime (Paterson and Bonomo, 2005). The rising prevalence of ESBL-producing *Enterobacteriaceae*, including *E. coli*, has led to a surge in infections globally, posing a significant threat to public health (Pitout and Laupland, 2008).

Historically, the introduction of extended spectrum cephalosporins in clinical practice in the 1970s was swiftly followed by reports of resistant strains of *Enterobacteriaceae* producing ESBLs (Livermore, 1995). The situation has only intensified over the years, with ESBL-resistant strains causing a substantial burden on healthcare systems worldwide. In the United States alone, the Centers for Disease Control and Prevention estimates an alarming 26,000 infections and 1,700 deaths annually attributed to ESBL-producing *Enterobacteriaceae* (CDC, 2024).

The specific subtype of ESBL-producing *E. coli*, those harboring CTX-M-type ESBLs, has gained prominence due to its association with both hospital-acquired and community infections (Cantón and Coque, 2006). Notably, these strains have been implicated in urinary tract infections and bacteremia, contributing significantly to the global epidemiology of ESBLs (Castanheira et al., 2021). The ability of CTX-M-producing *E. coli* to transfer resistance genes via plasmids further complicates the issue, as these mobile genetic elements can spread resistance rapidly across different bacterial populations and environments (Carattoli, 2009).

In recent years, the detection of CTX-M-producing *E. coli* in food animals, including poultry, and their products, as well as in pets and wild birds, has raised significant public health concerns (Dierikx et al., 2013; Ewers et al., 2012). The potential transmission of resistance genes through the food chain emphasizes the importance of studying AMR in food-producing animals. Poultry, in particular, plays a crucial role in this dynamic due to the high volume of antimicrobial usage in industry in some parts of the world, and the close contact between animals and humans (Landers et al., 2012).

In Bangladesh, where poultry farming is a vital economic activity, the issue of antimicrobial resistance in broiler chickens warrants urgent attention. The imprudent use of antimicrobials in poultry farming practices has likely contributed to the emergence and spread of resistant bacterial strains, including cefotaxime-resistant *E. coli* (CREC) (Islam et al., 2012). Despite the global implications, there is a scarcity of comprehensive studies examining the molecular epidemiology and resistance dynamics of these strains in Bangladeshi poultry farms. To bridge this gap in knowledge, we aim to determine the population structure, phylogeny, and patterns of antimicrobial resistance among the CREC isolated from broiler chicken farms in Bangladesh. By comparing our findings with a global collection of CREC, we seek to elucidate the interconnectedness of resistance profiles on a broader scale.

## Materials and Methods

### Study farms and sampling

A longitudinal study was conducted (Ahmed et al., 2020) with partial data previously published. From the same study, we isolated colistin-resistant and cefotaxime-resistant *E. coli* separately. Although the colistin-related results were published previously, all cefotaxime-associated work and isolates are included in this publication. Briefly, commercial broiler chicken farms in the Chattogram division of Bangladesh were selected. Farm owners were invited to participate based on new flock starting dates, a minimum flock size of 500 birds, and geographic representation. Pooled fecal samples were collected from each farm on day 1, day 15, and day 28 of production. Each pooled sample included five fresh fecal droppings from different flock areas. A trained veterinarian collected the samples and transported them to the laboratory on the same day in a cooling box. A questionnaire survey was conducted to gather epidemiological data, including bird strains, antimicrobial use, food and water sources, and farm biosecurity practices. Farm owners provided informed consent, and the study was approved by the Animal Ethical Committee of Chattogram Veterinary and Animal Sciences University (CVASU), Bangladesh.

### Bacterial isolation and identification

Cefotaxime-resistant *E. coli* (CREC) was isolated and quantified from fecal samples using the drop plate method (Miles et al., 1938) on MacConkey agar (MCA) (Oxoid, UK) with 4 mg/L cefotaxime, essentially as previously described (Græsbøll et al., 2017). Lactose-positive, red, non-mucoid colonies were counted, selected, and sub-cultured on Eosin Methylene Blue agar (EMB) (Scharlau, Spain) for biochemical confirmation. The species identity of *E. coli* was verified by standard biochemical properties and species-specific multiplex PCR targeting the *uidA* and *uspA* genes as described previously (Godambe et al., 2017). Amplified PCR products were separated via electrophoresis using 2% agarose gel (Sigma-Aldrich, USA) containing ethidium bromide (AMRESCO, USA) and visualized under UV light. The GeneRuler 100 bp Plus DNA Ladder (Thermo Fisher Scientific, USA) was used for standardization. Confirmed *E. coli* isolates were preserved at -80°C after subculture on Blood agar (BA) (Oxoid, UK).

### Phenotypic detection of ESBL

The phenotypic detection of extended-spectrum beta-lactamase (ESBL) producers was performed by double-disc diffusion synergy test (Kaur et al., 2013) using the commercially available Kit I and Kit III for ESBL identification (Himedia, India). Each kit includes cartridges containing antibiotic discs: cefotaxime (30 µg), cefotaxime/clavulanic acid (30/10 µg), ceftazidime (30 mcg), and ceftazidime/clavulanic acid (30/10 µg). The procedure involved preparing Mueller Hinton Agar (Oxoid, UK) plates and inoculating them with bacterial suspensions standardized to a 0.5 McFarland turbidity standard. Discs were applied aseptically and incubated at 35°C for 16–18 h. Zones of inhibition were measured and interpreted according to CLSI guidelines (CLSI, 2018). For ESBL detection, the presence of extended-spectrum beta-lactamase was confirmed if the inhibition zone increased by at least 5 mm for ceftazidime/clavulanic acid compared to ceftazidime alone, or by at least 3 mm for cefotaxime/clavulanic acid compared to cefotaxime alone. Confirmed ESBL-producing isolates were reported as resistant to all penicillins, cephalosporins, and aztreonam. Quality control was ensured using *E. coli* ATCC 25922.

### PCR for ESBL genes and minimum inhibitory concentration (MIC) determination for cefotaxime resistance

All isolates were screened by a multiplex PCR to detect nine ESBL genes (*bla*_TEM_, *PampC*, *bla_SHV_*, *bla*_CTX-M_, *bla_CMY-1_*, *bla_CMY-2_*, *bla_OXA-1_*, *bla_OXA-2_*, *bla_ACC-1_*) as described previously (Hasman et al., 2005; Miró et al., 2002; Olesen et al., 2004). *E. coli* NCTC 13846 was used as a control. GeneRuler 100 bp Plus DNA Ladder (Thermo Fisher Scientific, USA) was used as an external reference control. The MIC for cefotaxime was determined by the broth microdilution (BMD) method according to ISO-standard (20776-1). Pure cefotaxime (Sigma-Aldrich, USA) and cation-adjusted (Ca^2+^ and Mg^2+^) Mueller Hinton II Broth (Sigma-Aldrich, USA) was used in the BMD test. The quality control of the experiment was monitored by a resistant strain *E. coli* NCTC 13846 and a susceptible strain *E. coli* ATCC25922.

### Whole Genome Sequencing (WGS) and analysis

Twenty CREC isolates, representing various sampling times and farms, were selected for WGS. Genomic DNA was extracted using the DNeasy Blood and Tissue Kit (Qiagen, Germany). Sequencing libraries were prepared following Illumina protocols and paired-end sequencing was performed on the Illumina MiSeq platform (Illumina, USA) as previously described (Ahmed et al., 2020).

### Quality checking and de novo assembly

Sequencing read quality was assessed using FastQC (Galaxy Version 0.74+galaxy0). High-quality reads were de-novo assembled into draft genomes using the Unicycler assembly pipeline (Unicycler version 0.5.0), which integrates both Illumina and long reads for accurate assemblies. Assembly quality was evaluated using Quast (Quast Version 5.2.0).

### MLST typing and population structure analysis

Multi-locus sequence typing (MLST) was performed for all isolates using the NCBI PubMLST tool (https://github.com/tseemann/mlst). The command *mlst --csv assembled_genome* > *mlst.csv* was used, which automatically selects the appropriate scheme and outputs the sequence type (ST) and corresponding allelic information for each strain. This process involves analyzing seven housekeeping genes: *adk*, *fumC*, *gyrB*, *icd*, *mdh*, *purA*, and *recA*. The allelic profiles obtained from MLST were combined with metadata to explore the population structure using the GrapeTree tool (Zhou et al., 2018). In GrapeTree, the following steps and parameters were applied: launching the GrapeTree executable file, uploading the MLST profile (mlst_profile.txt), and uploading the metadata file.

### Global phylogeny

For a comprehensive global phylogenetic comparison, WGS data from 456 CREC strains from previous studies were included (Supplementary File 1). To identify potential CREC strains for the comparative study, we conducted a systematic search in PubMed that used whole-genome sequencing on cefotaxime resistant *E. coli*. The literature search was performed on 26 December 2023 using the following advanced search terms: ((*"cefotaxime-resistance"[Title/Abstract] OR "cefotaxime-resistant"[Title/Abstract] OR "cefotaxime resistant"[Title/Abstract] OR "cefotaxime resistance"[Title/Abstract]) AND ("*E. coli*"[Title/Abstract] OR "*Escherichia coli*"[Title/Abstract])) AND ("genome"[Title/Abstract] OR "WGS"[Title/Abstract] OR "genomic"[Title/Abstract] OR "Whole genome sequence"[Title/Abstract] OR "NGS"[Title/Abstract] OR "Next-generation sequencing"[Title/Abstract] OR "Illumina"[Title/Abstract] OR "Minion"[Title/Abstract] OR "Pacbio"[Title/Abstract]*). This search yielded 28 publications. However, after a full-text review, we identified 11 publications with complete metadata and publicly available WGS data. From these studies, we downloaded 456 whole-genome sequences, as detailed in Supplementary File 1. Phylogenetic analysis was conducted using Parsnp, a command-line tool designed for efficient microbial core genome alignment and single nucleotide polymorphism (SNP) detection. The *E. coli* str. K-12 substr. MG1655 was used as reference genome (Ecolik12_NC_000913.3.fasta), and the assembled genomes were aligned to this reference. The Parsnp tool (Kille et al., 2024) was executed with the command *parsnp -r reference.fasta -d assembled_genome -p 16 -c -x*, where the -r flag specifies the reference genome, the -d flag indicates the directory containing the assembled genomes, the -p 16 flag sets the number of threads for parallel processing, the -c flag enables the core genome alignment, and the -x flag excludes highly repetitive regions to avoid potential alignment issues. The resulting phylogenetic tree was visualized and annotated using the Interactive Tree Of Life (iTOL) tool (Letunic and Bork, 2021).

### In silico typing

Antimicrobial resistance (AMR) genes, chromosomal mutations associated with AMR, biocide, metal and stress resistance, and virulence profiles were identified using the AMRFinderPlus tool (https://www.ncbi.nlm.nih.gov/pathogens/antimicrobial-resistance/AMRFinder/). The analysis was performed using the command amrfinder -n "$fasta_file" -O Escherichia --plus --mutation_all "$output_dir/${filename_no_ext}_point_mut_report.txt" --name "$filename_no_ext" -o "$output_dir/${filename_no_ext}_output_file.txt" --ident_min 0.90 --coverage_min 0.70. This command processes each FASTA file, identifying AMR genes and mutations with a minimum identity threshold of 90% and a coverage threshold of 70%. The --plus option was used to include additional databases for a more comprehensive analysis. Results were output to specific files for each genome, with one file containing point mutation reports and another containing the overall AMR profile. Plasmid replicon identification was performed using the *staramr* tool (Bharat et al., 2022) available on the Galaxy platform (Galaxy Version 0.10.0+galaxy1). This tool scans genome assemblies against the PlasmidFinder (Carattoli et al., 2014) to identify Plasmid replicon.

## Results

### Sampling and study area

**Fig. 1.**
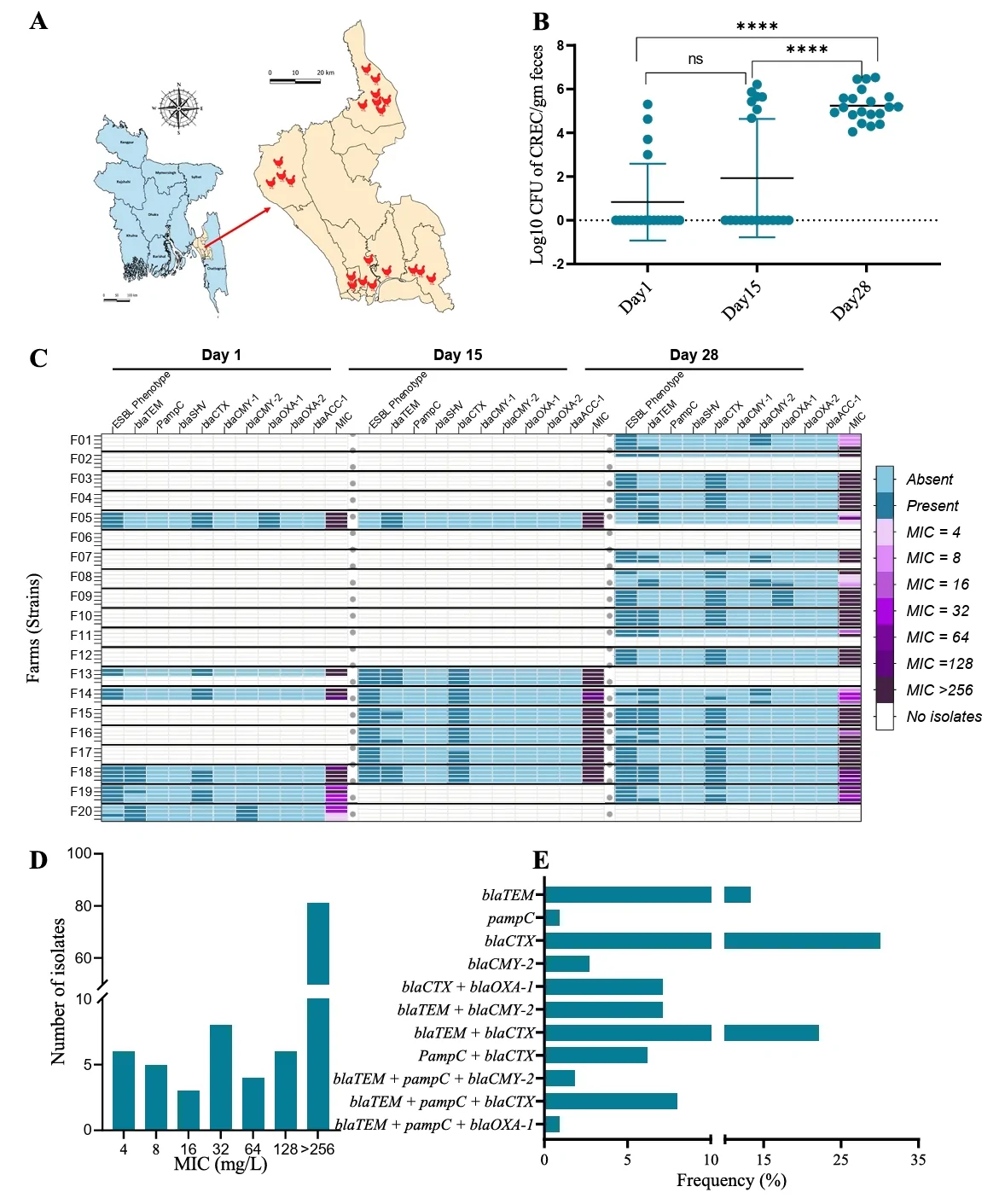
Cefotaxime-resistant *E. coli* (CREC) isolated from broiler chicken farms in Bangladesh. (A) Map of Bangladesh, showing the geographical location of the broiler chicken farms where fecal samples were collected, and CREC were isolated. (B) Average count of CREC per gram of fecal samples over three sampling time points. Asterisks denote statistical significance based on a two-tailed unpaired *t*-test between time points, where **** indicates *p*-value < 0.0001 and NS indicates non-significant (*P* > 0.05). (C) Detailed overview of the distribution of CREC strains across farms. Horizontal labels F01 to F20 denote farm IDs, separated by bold horizontal lines. Fecal samples were collected from each farm at three time points (Day 1, Day 15, and Day 28). Within each farm, our goal was to isolate four strains. Therefore, each rectangular box represents a single strain from that farm. Empty or white small rectangular boxes represent non-detectable CREC isolates for that particular farm and time point. The three large columns represent sampling times. Under each sampling time, individual columns represent ESBL phenotype based on the double-disc synergy test, PCR test results for the mentioned ESBL gene, and MIC value against cefotaxime. (D) Number of CREC strains according to MIC distribution. (E) Frequency of ESBL genotypes and combination groups.

A total of 60 pooled fecal samples were collected from 20 commercial broiler chicken farms across Bangladesh, as described in a previous study (Ahmed et al., 2020). The geographical locations and distances between the farms are shown in Fig. 1A. This sampling strategy ensured a representative understanding of CREC prevalence and distribution across different farming operations. The farms selected for this study were geographically dispersed across two administrative districts in the Chattogram division, with distances between farms ranging from 0.5 km to 80 km. Flocks sampled ranged in size from 800 to 4,000 birds, with a median size of 1,060 birds per farm. For further details on farm characteristics, antimicrobial usage, and sampling methodology, please refer to the original study by Ahmed et al. (2020).

### Increased resistance against cefotaxime observed at the end of a production cycle

Our study observed a significant increase in cefotaxime resistance over the course of a production cycle. Quantitative measurements revealed a notable rise in CREC counts from day 1 to day 28. Initially, the average count of CREC at day 1 was 0.83 ± 1.75 log10 CFU/g feces, which increased to 1.93 ± 2.71 log10 CFU/g feces by day 15, and further surged to 5.24 ± 0.72 log10 CFU/g feces by day 28 (Fig. 1B). This data indicates the progressive amplification of resistant bacteria as the production cycle advances. At the beginning of the production cycle, only 20% (4/20) of the farms had detectable levels of CREC. By day 15, this frequency increased to 35% (7/20), and by day 28, 85% (17/20) farms tested positive for CREC (Fig. 1C). This trend indicates either a rapid dissemination and establishment of resistance within a relatively short time frame or that CREC are present in all farms a very low levels (below detection level) at the beginning of the cycle, and that numbers increase as a function of the management during the production.

### ESBL phenotype, MIC distribution and PCR based genotyping

A detailed analysis of the antimicrobial resistance profiles of all CREC isolates was conducted. Not only did the frequency and CFU count of CREC increase over time, but we also detected a higher prevalence of resistance genes throughout the production cycle. On day 1, 86% (18/21) of the CREC isolates were confirmed as ESBL producers by double-disc diffusion synergy test. This proportion remained consistent, with 86% (24/28) on day 15 and 84% (51/61) on day 28, indicating a sustained presence of resistant phenotypes (Fig. 1C). Cefotaxime MIC distribution further supported these findings, showing a higher tendency towards elevated MIC values in the day 28 samples. The overall MIC distribution of CREC isolates varied from 4 to >256 mg/L, with more than 70% of the isolates exhibiting an MIC of ≥ 256 mg/L against cefotaxime (Fig. 1D). This high MIC value indicates a substantial level of the resistance among the isolates. To understand the genetic basis of resistance, we characterized the CREC strains using PCR to target nine different ESBL-related genes. The distribution of these genes is presented in Fig. 1C. The ESBL genotype *bla*_CTX-M_ was the most frequently detected resistance gene, found in 30% of the isolates. This was followed by the *bla*_TEM_ + *bla*_CTX-M_ genotype, present in 22% of the isolates (Fig. 1E).

### Whole genome sequencing and genome assembly

In this study, twenty CREC strains were selected for whole-genome sequencing. The selection was based on representation across three sampling time points, origin from various farms, and the highest MIC values for cefotaxime. Additionally, 456 whole genome sequences of CREC strains from a global collection were included for comparative analysis. This global collection represents isolates from nine countries (China [n=110], Czech Republic [n=76], Guadeloupe [n=28], Ireland [n=6], Mexico [n=4], Spain [n=66], The Netherlands [n=109], USA [n=1], and Zambia [n=56]) and 12 different sources, as detailed in Supplementary File 1. The global strains were isolated using selective isolation procedures similar to those employed in our study, ensuring consistency in comparative analysis. We used Unicycler assembler, to produce and assemble draft genomes for the 20 *E. coli* strains from our study. The same assembly approach was applied to the raw sequencing data from the global collection where available, while pre-assembled genomes those are already assembled by the original study were directly used for downstream analysis. The resulting draft genomes from our 20 strains had a median assembly length of 5.05 Mb. The average N50 of the assembled contigs was 119 kb for our study strains and 165 kb when including the global collection (Supplementary File 2). This indicates a higher contiguity in the global collection assemblies, possibly due to differences in sequencing technologies. The median number of contigs per assembly was 125 for our study strains and 100 for all isolates, including the global collection (Supplementary File 2).

### Genomic determinants of AMR and virulence in cefotaxime resistant *E. coli*

**Fig. 2.**
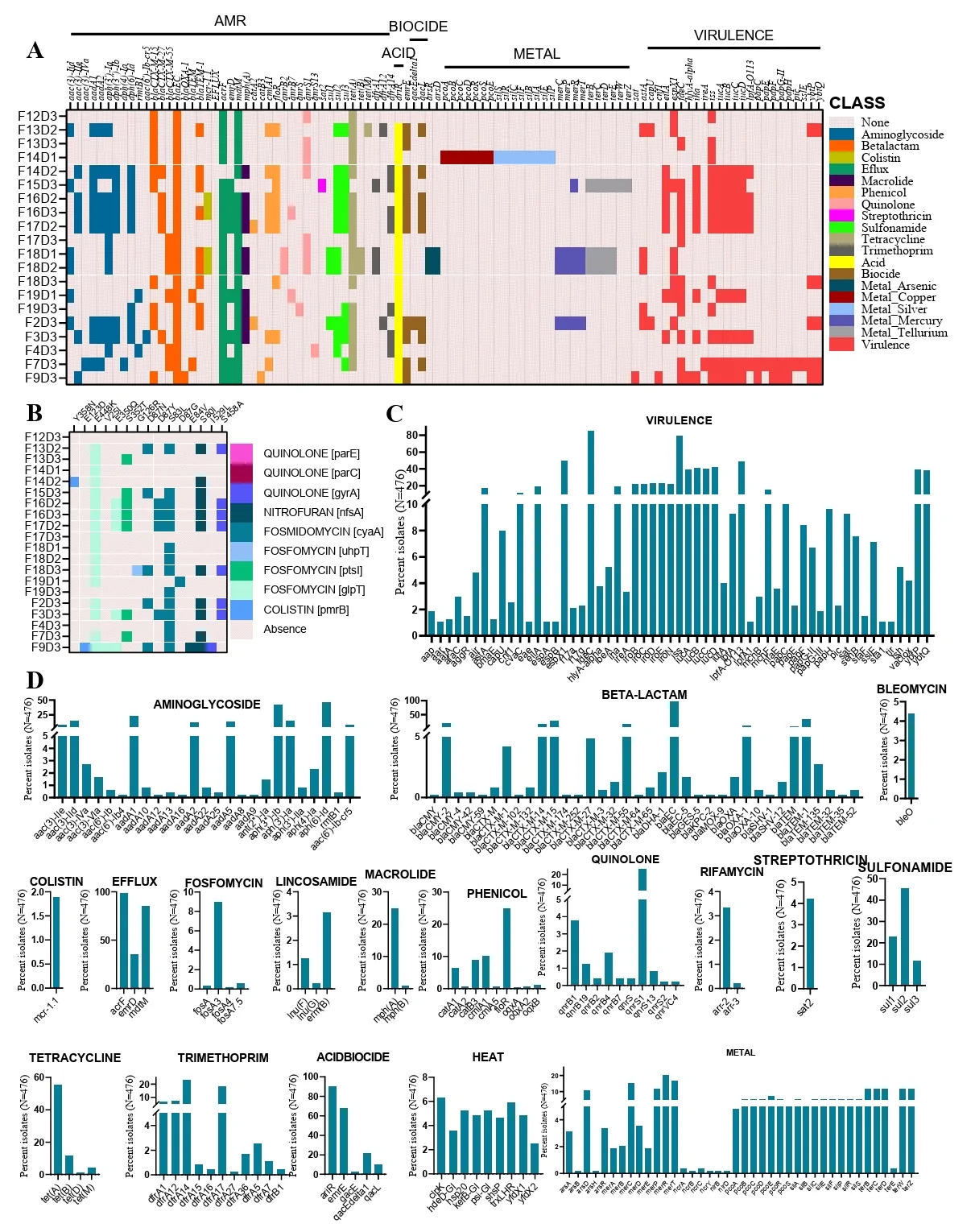
Genomic characterization of cefotaxime-resistance in *E. coli*. (A) Heatmap showing antimicrobial resistance determinants, acid, biocide, and metal resistance genes, and virulence genes of 20 isolates collected from broiler chicken farms in Bangladesh. Row headers represent strain IDs. (B) Heatmap representing chromosomal point mutations in the CREC genome. Colors indicate amino acid substitutions at the corresponding mutation sites. Rows and columns represent isolates and mutated genes, respectively. (C) Frequency of virulence genes identified among the 476 CREC strains. (D) Frequency of isolates carrying different AMR, metal resistance, stress, and biocide resistance genes among the 476 CREC strains.

In our study of 20 CREC strains, we identified a total of 40 different AMR determinants (Fig. 2A). Among the resistance determinants, we detected three types of ESBL genes. The most common was *bla*_CTX-M-15_, found in 10 out of 20 strains, followed by *bla*_CTX-M-55_ in 6 strains, and *bla*_CTX-M-27_ in 5 strains. Notably, two isolates co-harbored two of these ESBL genes (Fig. 2A). The most abundant AMR gene was *bla*_EC_, found in 20 out of 32 strains. Notably, all 20 strains carried multiple acquired AMR genes, ranging from a minimum of six to a maximum of 20 genes per strain (Fig. 2A). Beyond antimicrobial resistance, we also explored resistance genes related to acids, biocides, and metals. We detected three acid resistance genes, three biocide resistance genes, and 29 metal resistance genes among the 20 strains. The metal resistance genes provided resistance to arsenic, copper, silver, mercury, and tellurium. In total, 24 virulence genes were identified in the genomes of these CREC strains (Fig. 2A). The most prevalent virulence determinants were *iss* (Increased Serum Survival) and *fdeC* (Intimin-like adhesin), found in 65% and 60% of the isolates, respectively. Interestingly, a significant portion of the isolates (60%) harbored at least five virulent genes. We further investigated the genomic mechanisms underlying certain resistance phenotypes by analyzing point mutations in relevant genes. Our analysis revealed 15 known point mutations associated with resistance to fluoroquinolones, nitrofurans, fosfomycin, and colistin (Fig. 2B, Supplementary File 3).

To place our findings in a global context, we compared the AMR and virulence profiles of our 20 isolates with those of CREC strains worldwide. The comparative analysis showed that, similar to our study, the most frequent virulence gene globally was *fdeC*, present in 85% of the 476 strains analyzed, followed by *iss*, found in 80% of the strains (Fig. 2C). Globally, 40% of the strains carried at least two aminoglycoside resistance genes, with the most frequent being *aph(6)-Id* and *aph(3'')-Ib* (Fig. 2D). Notably, we detected 17 different types of ESBL genes among the global collection of strains. The most frequent ESBL gene, like our study, was *bla*_CTX-M-15_, found in 113 out of 476 strains, followed by *CMY-2* in 86 strains, and both *bla*_CTX-M-14_ and *bla*_CTX-M-15_ in 67 strains (Supplementary File 3). We also detected 36 beta-lactam resistance genes across the global strain collection, including our study, with *bla*_EC_ and *bla*_TEM_-1 being the most common, present in 98% and 32% of strains, respectively (Fig. 2D). Additionally, notable resistance genes across different antimicrobial classes included *qnrS1* for quinolone resistance (20%), *sul2* for sulfonamide resistance (45%), and *tetA* for tetracycline resistance (56%). The frequency of other resistance genes within their respective antimicrobial classes is illustrated in Fig. 2D.

### Population structure and sequence types of CREC strains

**Fig. 3.**
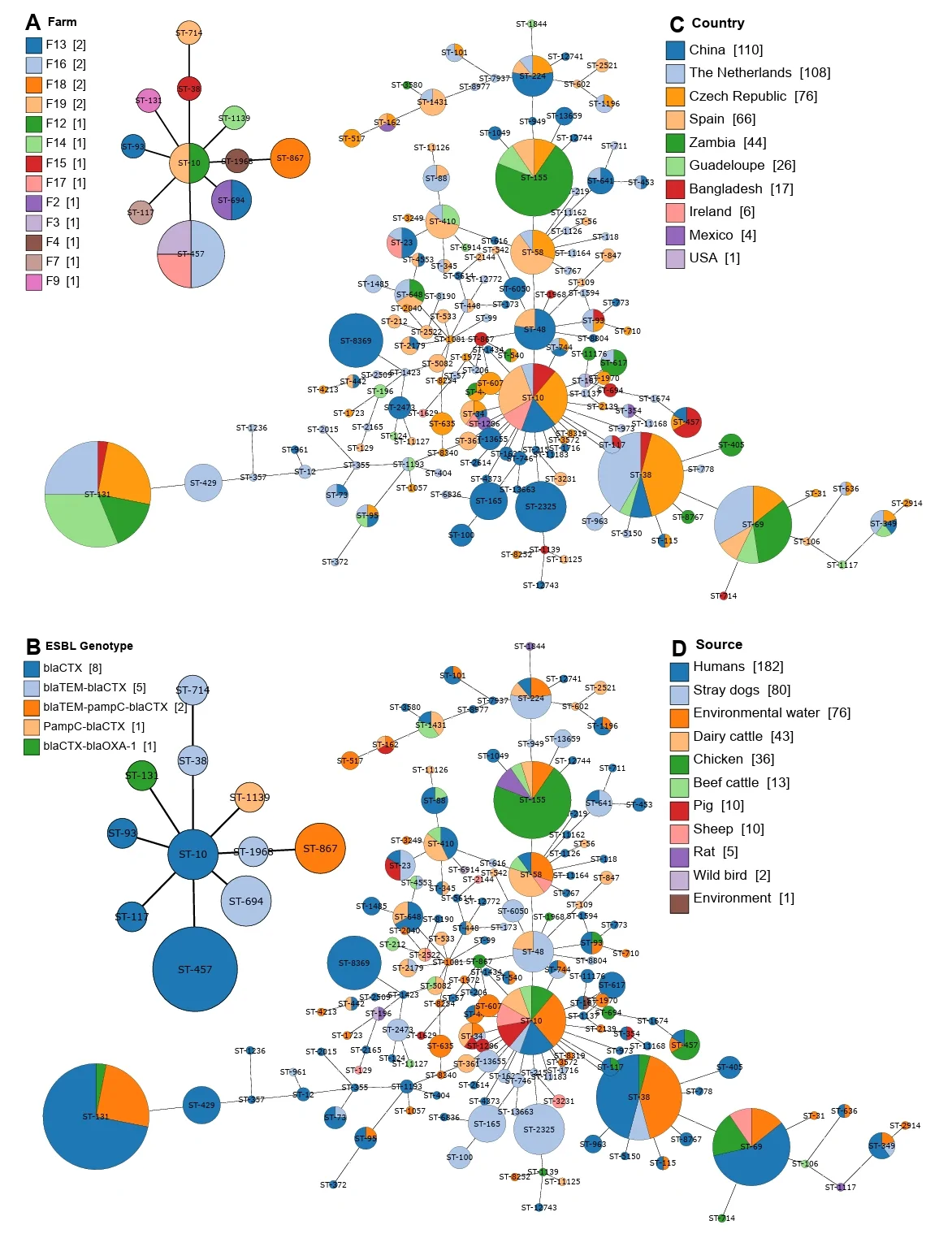
Minimal spanning tree showing the population structure of cefotaxime-resistant *E. coli*. (A) Population structure of 20 CREC strains isolated from broiler chicken farms in Bangladesh, annotated based on the farm of origin. (B) Population structure of the same 20 CREC strains, annotated based on the ESBL genotype. (C) Population structure of 476 CREC strains from a global collection, annotated based on the country of origin. (D) Population structure of the same 476 CREC strains, annotated based on the source of origin. Each circle corresponds to a Sequence Type (ST), with its size reflecting the number of isolates associated with that particular ST. The length of the connecting line between two circles is proportional to the dissimilarity in the number of MLST loci between the two respective STs.

In our analysis of 20 CREC strains, we identified a total of 11 sequence types (STs). The most frequent sequence types were ST457 (n=4), followed by ST694 (n=2) and ST867 (n=2) (Fig. 3A). The population structure analysis, conducted using a minimum spanning tree, revealed that some sequence types were shared across different farms, even those distantly located within the study area (Fig. 1A). For instance, ST457 was found in three distinct farms, suggesting the widespread geographical presence of these isolates in chicken farms. Moreover, the population structure analysis indicated an association between ST type and the type of ESBL gene carried in the strains (Fig. 3B). This highlights the potential clonal expansion of specific resistant strains within the region.

Expanding our comparison to a global scale, we identified a total of 157 sequence types among 476 CREC strains. Among these, the most dominant sequence types were ST131 and ST38, found in 32 and 24 strains, respectively. The global population structure analysis showed that ST10 was the most geographically diverse sequence type, detected in six countries, including China, the Czech Republic, the Netherlands, Ireland, and Bangladesh (Fig. 3C). This widespread distribution emphasizes the global dissemination of specific CREC strains. Additionally, we observed country-specific distributions of CREC sequence types. For example, ST155 was dominant in Zambia, while ST-38 was prevalent in the Czech Republic (Fig. 3C). We further analyzed the population structure to detect source-specific clustering of sequence types. Similar to its global distribution, ST10 was found in the most heterogeneous sources, spanning eight different environments. The most dominant sequence type, ST131, was primarily detected in humans and environmental water (Fig. 3D). However, some sequence types exhibited high source specificity; for instance, ST8369 was detected only in humans, and ST2325 was found exclusively in stray dogs. This analysis highlights the complex and dynamic population structure of CREC strains, emphasizing the need for continuous surveillance to understand the spread and evolution of antimicrobial resistance in both local and global contexts.

### Comparative phylogeny and plasmid genotypes

**Fig. 4.**
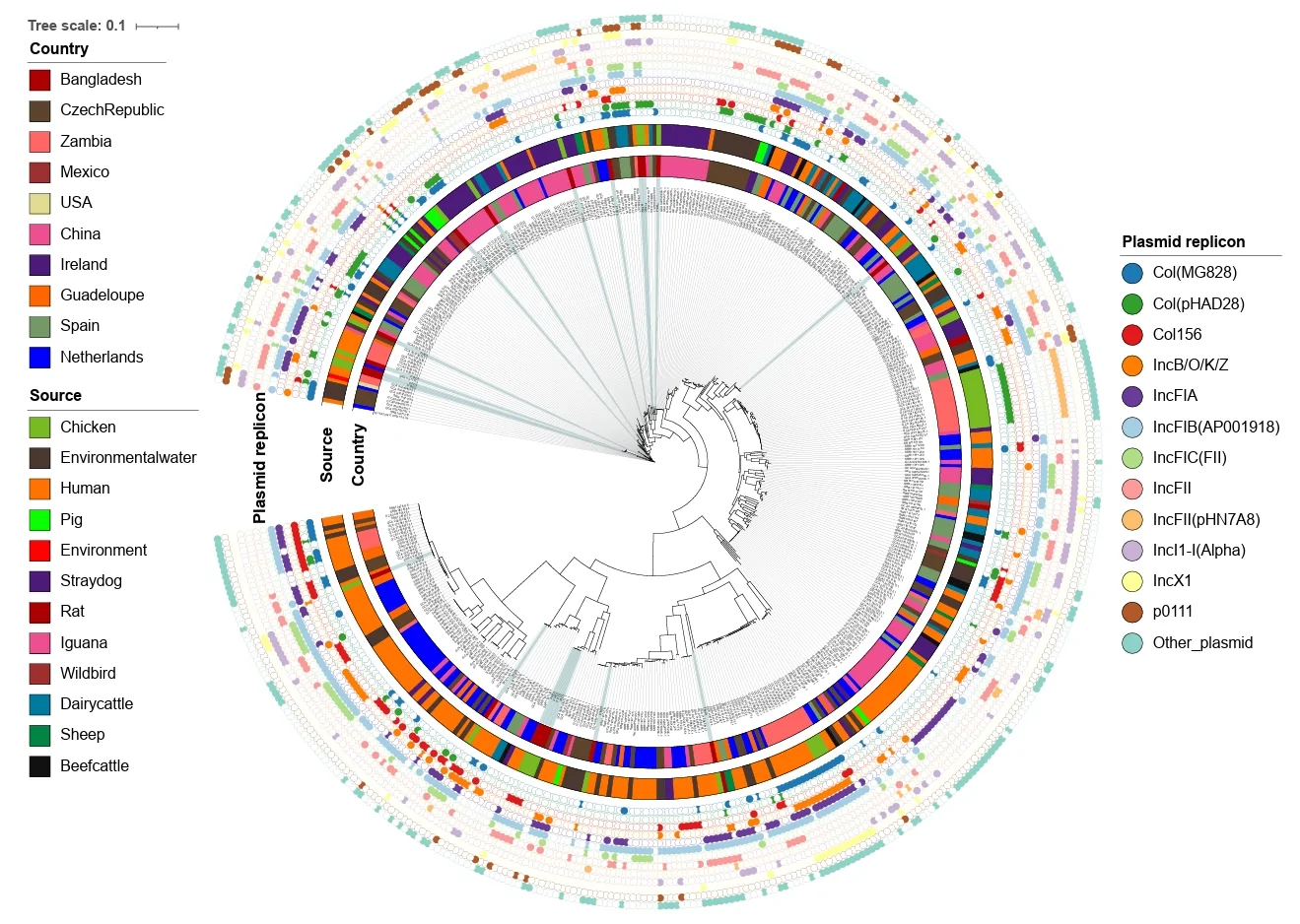
Maximum-likelihood phylogenetic tree based on concatenated core genome sequences of 476 cefotaxime-resistant *E. coli* isolates. The tree is based on 61,495 SNPs originated from core-genome analysis, focusing on conserved regions across the genomes excluding the recombinant regions. All assembled genomes of *E. coli* were aligned against *E. coli* K12 reference genome (NC_000913.3). The outer annotation ring represents country, source, and plasmid replicon types.

The whole genome sequences of the 20 strains identified in the current study were compared to the global collection (Supplementary File 1) by SNP analyses. A total of 61,495 core SNPs were detected among the 476 isolates, revealing diverse phylogenetic clustering. Phylogenetic analysis of the strains from this study shows that they form multiple small clusters with other global strains, indicating their widespread geographical presence. Notably, the largest cluster from this study, containing four strains, forms a subcluster with strains primarily from the Netherlands, all of which were isolated from humans. Several other clusters demonstrate close relationships with isolates of human, animal, and environmental origins. This emphasizes the importance of applying a One Health approach to mitigate and minimize the spread of antimicrobial-resistant bacterial strains across different sources, particularly from animals to humans (Fig. 4).

To investigate the diversity of mobile genetic elements, plasmid replicon typing was performed using WGS data. We identified 25 different plasmid replicons in the 20 CREC isolates from this study, with 17 strains showing the presence of at least two plasmid replicons. The most frequent replicon type was IncFIB(AP001918), found in 12 strains, followed by Col(pHAD28) in 10 strains. Other dominant plasmid replicon types included IncFII (7/20), Col156 (6/20), and IncN (6/20) (Supplementary File 4).

At the global level, 63 plasmid replicon types were identified among the 476 CREC strains. The most common types were IncFIB(AP001918) found in 274 strains, followed by IncFII present in 147 strains. Other dominant types included Col(MG828) (n=129), IncFIA (n=127), and IncI1-I(Alpha) (n=112) (Fig. 4).

## Discussion

The present study aimed to elucidate the prevalence, resistance patterns, and genetic characteristics of CREC isolated from broiler chicken farms in Bangladesh, and to compare these findings with a global collection of CREC strains. Our results revealed important understandings into the dissemination and genetic basis of antimicrobial resistance in CREC.

Our findings demonstrate a notable increase in cefotaxime resistance over the course of a production cycle. Initial CREC counts were low on day 1, but they escalated significantly by day 28, with almost all farms testing positive for CREC by the end of the production cycle. This trend highlights the rapid dissemination and amplification of resistance within farms during a relatively short period, likely facilitated by selective pressure from antibiotic use in poultry farming (Ahmed et al., 2020). This is consistent with other studies that have shown similar trends in the increase of antimicrobial-resistant bacteria in livestock over time (Van Boeckel et al., 2015). However, it is difficult to ascertain whether the increased resistance at the end of the production cycle was associated with increased use of cefotaxime, as only one of the studied farms used cephalosporin (Ahmed et al., 2020). To further investigate the potential association, we performed a logistic regression analysis using the overall antimicrobial use data, including amoxicillin use, to determine if there is any association with the ESBL resistance phenotype among the cefotaxime-resistant isolates. Our analysis did not detect any significant association between overall antibiotic use and amoxicillin use with the frequency of the ESBL resistance phenotype. This aligns with the findings of other studies which suggested that resistance patterns are often multifactorial and cannot be attributed solely to the use of a single antibiotic class (Kümmerer, 2004; Tang et al., 2017). The high prevalence of CREC in broiler chicken farms, despite no use of cephalosporin drugs in almost all farms, is alarming, as it suggests that resistance genes can rapidly disseminate across different regions, facilitated by the movement of animals, people, and agricultural products.

The phylogenetic analysis revealed diverse clustering of CREC strains from this study with those from other countries, indicating widespread geographical presence of many clones. Notably, the largest cluster from this study formed a subcluster with strains primarily from the Netherlands, all isolated from humans. This close relationship between strains from different sources emphasizes the importance of the One Health approach in addressing antimicrobial resistance, as it affects human, animal, and environmental health (Fig. 4). Our comparative phylogenetic analysis with a global collection of CREC strains illustrates the interconnectedness of antimicrobial resistance across borders. Similar findings have been reported in other studies, which have demonstrated the global dissemination of resistant strains through trade and travel (Bokhary et al., 2021). The presence of CREC strains in both human and animal sources highlights the role of zoonotic transmission in the spread of antimicrobial resistance. This aligns with previous reports that have documented the transfer of resistant bacteria from animals to humans, emphasizing the need for integrated surveillance and control measures (Cao et al., 2022).

Our study identified 25 plasmid replicons among the 20 CREC isolates, with IncFIB(AP001918) being the most frequent. This replicon type was also predominant in the global collection, suggesting its significant role in the dissemination of resistance genes. Interestingly, this plasmid replicon has been reported as a dominant type in MDR *E. coli* strains globally (Adator et al., 2020; Kidenya et al., 2023; Medina-Pizzali et al., 2022; Wang et al., 2024). This suggests it may play a significant role in the dissemination of AMR genes. For example, studies have shown that the presence of this replicon correlates with increased resistance to multiple antibiotics, contributing to the spread of multidrug-resistant strains (Kidenya et al., 2023). However, our analysis did not detect any colocalization of this plasmid replicon with any of the ESBL genes in our strains, as they were found on different contigs (Supplementary File 1). This indicates that while the plasmid replicon is prevalent and associated with AMR dissemination, its relationship with specific ESBL genes may vary across different bacterial populations and environmental contexts. Further research is needed to elucidate the mechanisms underlying this variability and to explore potential strategies for mitigating the spread of resistance genes. The presence of multiple plasmids in most isolates indicates the complexity of resistance mechanisms and the potential for horizontal gene transfer, further complicating efforts to control the spread of resistance (Supplementary File 3). Previous studies have highlighted the role of plasmids in the spread of resistance genes, with certain plasmid types being associated with multi-drug resistance in various bacterial species (Li et al., 2019).

The detection of a high prevalence of ESBL phenotype and resistance genes, such as *bla*_CTX-M_, highlights the genetic basis for cefotaxime resistance in CREC. The constant presence of these genes throughout the production cycle suggests persistent selection pressure and stable maintenance of resistance genes in the bacterial population (Fig. 1C). The *bla*_CTX-M_ gene family, particularly *bla*_CTX-M-15_, has been identified as a major contributor to ESBL production and cefotaxime resistance in *E. coli* (Cantón et al., 2012). *bla*_CTX-M-15_ is notably significant for human health due to its widespread occurrence and potent ability to confer resistance to third-generation cephalosporins, making infections difficult to treat (Woerther et al., 2013). Our findings align with global trends, where *bla*_CTX-M-15_ has emerged as the most prevalent ESBL gene in both clinical and agricultural settings (Woerther et al., 2013). This prevalence highlights the potential zoonotic transfer of resistant strains between animals and humans. The transmission of *bla*_CTX-M-15_ from livestock to humans can occur through direct contact or via the food chain, illustrating the interconnectedness of animal and human health (Adator et al., 2020). It is important to consider that the global collection of strains might be biased due to the overrepresentation of strains from certain regions or specific environments, which can affect the generalizability of our results. For instance, regions with intensive agricultural practices or high antibiotic usage may report higher prevalence rates of certain resistance genes (Kidenya et al., 2023). This bias needs to be taken into account when interpreting our findings and comparing them to global data.

Our study emphasizes the need for regular monitoring and surveillance to understand the spread of AMR between humans and animals. Although the farms we studied do not use cephalosporins, our data shows increasing cefotaxime resistance towards the end of the production cycle. This suggests that resistance is being selected and accumulated. Continuous surveillance is essential to track these resistance patterns and inform policy decisions.

Despite the comprehensive nature of this study, certain limitations need to be acknowledged. The sample size was limited to broiler chicken farms in Bangladesh, which may not fully capture the diversity of CREC in other regions or farming systems. Additionally, while the study provides valuable insights into the genetic basis of resistance, functional validation of the identified resistance genes and plasmids was beyond the scope of this research. Another limitation is the reliance on selective isolation methods, which may bias the detection towards certain resistant strains while potentially missing others. Future studies should consider employing broader sampling strategies to obtain a more comprehensive understanding of resistance patterns.

## Conclusion

In conclusion, our study provides a detailed analysis of the prevalence, phylogenetic relationships, and genetic characteristics of cefotaxime-resistant *E. coli* from broiler chicken farms in Bangladesh. The findings demonstrate the rapid dissemination of resistance within a production cycle, the widespread geographical distribution of similar strains, and the complex genetic mechanisms underlying resistance. These findings highlight the critical need for integrated and global strategies to combat antimicrobial resistance. Our study contributes to the growing body of evidence supporting the One Health approach as essential for mitigating the spread of antimicrobial-resistant bacteria and protecting public health.

## Data Availability

All data are incorporated in this manuscript and additional data can be requested to the corresponding authors.

## Supplementary Information

Supplementary File 1. Study characteristics of all included strains.

Supplementary File 2. Output of AMRfinder plus

Supplementary File 3. Plasmid replicon typing output

Supplementary File 4. Genome assembly quality control summary

## References

[b1-jm-2412009] Adator EH, Walker M, Narvaez-Bravo C, Zaheer R, Goji N (2020). Whole genome sequencing differentiates presumptive extended spectrum beta-lactamase producing *Escherichia coli* along segments of the One Health continuum. Microorganisms.

[b2-jm-2412009] Ahmed S, Das T, Islam MZ, Herrero-Fresno A, Biswas PK (2020). High prevalence of *mcr-1*-encoded colistin resistance in commensal *Escherichia coli* from broiler chicken in Bangladesh. Sci Rep.

[b3-jm-2412009] Bharat A, Parmley EJ, Deckert A, Avery BP, Carson CA (2022). Correlation between phenotypic and *in silico* detection of antimicrobial resistance in *Salmonella enterica* in Canada using Staramr. Microorganisms.

[b4-jm-2412009] Bokhary H, Pangesti KNA, Rashid H, Abd El Ghany M, Hill-Cawthorne GA (2021). Travel-related antimicrobial resistance: A systematic review. Trop Med Infect Dis.

[b5-jm-2412009] Cantón R, Coque TM (2006). The CTX-M beta-lactamase pandemic. Curr Opin Microbiol.

[b6-jm-2412009] Cantón R, González-Alba JM, Galán JC (2012). CTX-M enzymes: origin and diffusion. Front Microbiol.

[b7-jm-2412009] Cao H, Bougouffa S, Park TJ, Lau A, Tong MK (2022). Sharing of antimicrobial resistance genes between humans and food animals. mSystems.

[b8-jm-2412009] Carattoli A (2009). Resistance plasmid families in *Enterobacteriaceae*. Antimicrob Agents Chemother.

[b9-jm-2412009] Carattoli A, Zankari E, Garcìa-Fernandez A, Larsen M, Lund O (2014). PlasmidFinder and pMLST: *in silico* detection and typing of plasmids. Antimicrob Agents Chemother.

[b10-jm-2412009] Castanheira M, Simner PJ, Bradford PA (2021). Extended-spectrum β-lactamases: an update on their characteristics, epidemiology and detection. JAC Antimicrob Resist.

[b13-jm-2412009] Dierikx C, van der Goot J, Fabri T, van Essen-Zandbergen A, Smith H (2013). Extended-spectrum-β-lactamase- and AmpC-β-lactamase-producing *Escherichia coli* in Dutch broilers and broiler farmers. J Antimicrob Chemother.

[b14-jm-2412009] Ewers C, Bethe A, Semmler T, Guenther S, Wieler LH (2012). Extended-spectrum β-lactamase-producing and AmpC-producing *Escherichia coli* from livestock and companion animals, and their putative impact on public health: a global perspective. Clin Microbiol Infect.

[b15-jm-2412009] Godambe LP, Bandekar J, Shashidhar R (2017). Species specific PCR based detection of *Escherichia coli* from Indian foods. 3 Biotech.

[b16-jm-2412009] Græsbøll K, Damborg P, Mellerup A, Herrero-Fresno A, Larsen I (2017). Effect of tetracycline dose and treatment mode on selection of resistant coliform bacteria in nursery pigs. Appl Environ Microbiol.

[b17-jm-2412009] Hasman H, Mevius D, Veldman K, Olesen I, Aarestrup FM (2005). β-Lactamases among extended-spectrum β-lactamase (ESBL)-resistant *Salmonella* from poultry, poultry products and human patients in The Netherlands. J Antimicrob Chemother.

[b18-jm-2412009] Islam MA, Talukdar PK, Hoque A, Huq M, Nabi A (2012). Emergence of multidrug-resistant NDM-1-producing gram-negative bacteria in Bangladesh. Eur J Clin Microbiol Infect Dis.

[b19-jm-2412009] Kaur J, Chopra S, Mahajan G (2013). Modified double disc synergy test to detect ESBL production in urinary isolates of *Escherichia coli* and *Klebsiella pneumoniae*. J Clin Diagn Res.

[b20-jm-2412009] Kidenya BR, Mboowa G, Sserwadda I, Kanyerezi S, Nakafu E (2023). Virulence genes and plasmid replicon profiles of selected β-lactamase-producing *Acinetobacter baumannii* from orthopaedic patients and the environment in a tertiary referral hospital in Tanzania, East Africa. J Hosp Infect.

[b21-jm-2412009] Kille B, Nute MG, Huang V, Kim E, Phillippy AM (2024). Parsnp 2.0: scalable core-genome alignment for massive microbial datasets. Bioinformatics.

[b22-jm-2412009] Kümmerer K (2004). Resistance in the environment. J Antimicrob Chemother.

[b23-jm-2412009] Landers TF, Cohen B, Wittum TE, Larson EL (2012). A review of antibiotic use in food animals: perspective, policy, and potential. Public Health Rep.

[b24-jm-2412009] Laxminarayan R, Duse A, Wattal C, Zaidi AKM, Wertheim HFL (2013). Antibiotic resistance—the need for global solutions. Lancet Infect Dis.

[b25-jm-2412009] Letunic I, Bork P (2021). Interactive Tree Of Life (iTOL) v5: an online tool for phylogenetic tree display and annotation. Nucleic Acids Res.

[b26-jm-2412009] Li Q, Chang W, Zhang H, Hu D, Wang X (2019). The role of plasmids in the multiple antibiotic resistance transfer in ESBLs-producing *Escherichia coli* isolated from wastewater treatment plants. Front Microbiol.

[b27-jm-2412009] Livermore DM (1995). β-Lactamases in laboratory and clinical resistance. Clin Microbiol Rev.

[b28-jm-2412009] Medina-Pizzali ML, Venkatesh A, Riveros M, Cuicapuza D, Salmon-Mulanovich G (2022). Whole-genome characterisation of ESBL-producing *E. coli* isolated from drinking water and dog faeces from rural Andean households in Peru. Antibiotics (Basel).

[b29-jm-2412009] Miles AA, Misra SS, Irwin JO (1938). The estimation of the bactericidal power of the blood. J Hyg (Lond).

[b30-jm-2412009] Miró E, Navarro F, Mirelis B, Sabaté M, Rivera A (2002). Prevalence of clinical isolates of *Escherichia coli* producing inhibitor-resistant β-lactamases at a university hospital in Barcelona, Spain, over a 3-year period. Antimicrob Agents Chemother.

[b31-jm-2412009] Olesen I, Hasman H, Aarestrup FM (2004). Prevalence of β-lactamases among ampicillin-resistant *Escherichia coli* and *Salmonella* isolated from food animals in Denmark. Microb Drug Resist.

[b32-jm-2412009] Paterson DL, Bonomo RA (2005). Extended-spectrum β-lactamases: a clinical update. Clin Microbiol Rev.

[b33-jm-2412009] Pitout JDD, Laupland KB (2008). Extended-spectrum β-lactamase-producing *Enterobacteriaceae*: an emerging public-health concern. Lancet Infect Dis.

[b34-jm-2412009] Tang KL, Caffrey NP, Nóbrega DB, Cork SC, Ronksley PE (2017). Restricting the use of antibiotics in food-producing animals and its associations with antibiotic resistance in food-producing animals and human beings: a systematic review and meta-analysis. Lancet Planet Health.

[b35-jm-2412009] Van Boeckel TP, Brower C, Gilbert M, Grenfell BT, Levin SA (2015). Global trends in antimicrobial use in food animals. Proc Natl Acad Sci USA.

[b36-jm-2412009] Van Boeckel TP, Glennon EE, Chen D, Gilbert M, Robinson TP (2017). Reducing antimicrobial use in food animals. Science.

[b37-jm-2412009] Wang L, Zhang R, Li J, Xu Y, Chen H (2024). Whole-genome sequencing of an *Escherichia coli* ST69 strain harboring *bla*_CTX-M-27_ on a hybrid plasmid. Infect Drug Resist.

[b38-jm-2412009] Woerther PL, Burdet C, Chachaty E, Andremont A (2013). Trends in human fecal carriage of extended-spectrum β-lactamases in the community: toward the globalization of CTX-M. Clin Microbiol Rev.

[b39-jm-2412009] Zhou Z, Alikhan NF, Sergeant MJ, Luhmann N, Vaz C (2018). GrapeTree: visualization of core genomic relationships among 100,000 bacterial pathogens. Genome Res.

